# Diabetes Distress in People at Risk for Diabetes‐Related Foot Disease in Primary Health Care

**DOI:** 10.1002/jfa2.70194

**Published:** 2026-07-29

**Authors:** Thallita Cláudia Moraes Barbosa, Letícia Eugênio Mota, Maria Gabriela Campos Nunes, Laura Andrade Pinto, Letícia Alves Rocha, Patricia Peres de Oliveira, Vinicius Silva Belo, Daniel Nogueira Cortez

**Affiliations:** ^1^ Graduate Program in Nursing Federal University of São João del‐Rei/Central‐West Campus Divinopolis Brazil; ^2^ Nursing Department Federal University of São João del‐Rei/Central‐West Campus Divinopolis Brazil; ^3^ Graduate Program in Health Sciences Federal University of São João del‐Rei/Central‐West Campus Divinopolis Brazil

**Keywords:** diabetes mellitus, diabetic foot, primary health care, psychological, risk factors, stress

## Abstract

**Introduction:**

Diabetes‐related distress (DRD) is exacerbated in people at risk of Diabetes‐related Foot Disease (DFD); however, knowledge on this topic is still limited, especially in Primary Health Care (PHC), which focuses on prevention and monitoring. This study evaluated DRD and its associated factors in PHC individuals at risk of developing DFD, aiming to support management strategies to reduce complications.

**Method:**

Cross‐sectional study with 1563 individuals with diabetes mellitus (DM) registered in PHC services in Brazil. Participants were stratified according to their risk of DFD, based on the criteria from the International Working Group on the Diabetic Foot. The DRD was measured using the Problem Areas in Diabetes (B‐PAID) scale, considering a score ≥ 40 as indicative of high distress. Sociodemographic, clinical, and behavioral variables were collected and analyzed using Gamma regression on the software *Statistical Package for the Social Sciences* (SPSS 26.0).

**Results:**

Among 1563 people with DM, 39.8% were at risk for DFD. In the multiple regression model, there is notable increase in DRD associated with greater difficulty in caring for the feet (*β* = 1.129; 1.014–1.257), loss of protective sensation (*β* = 1.311; 1.110–1.549), and poor glycemic control (*β* = 0.850; 0.761–0.951).

**Conclusion:**

Sociodemographic and clinical factors, particularly loss of protective sensation in the feet, difficulty in foot care, and inadequate control of glycated hemoglobin, are associated with greater DRD in people at risk for DFD. Primary health care stands out as a strategic space for integrating psychosocial support and clinical management, ensuring that preventive measures are implemented and resulting complications are avoided or detected early.

## 1 Introduction

Diabetes mellitus (DM) is a chronic condition that requires ongoing care and multifactorial risk reduction strategies, as well as glycemic control [1]. Among its complications, Diabetes‐Related Foot Disease (DFD) stands out for its high prevalence and severity, with manifestations such as peripheral neuropathy, peripheral arterial disease, infections, ulcers, neuro‐osteoarthropathy, gangrene or amputation, which can lead to individuals' physical, functional, and emotional limitations [2, 3]. The disease chronicity, the threat of complications, and the social burden contribute significantly to diabetes‐related distress (DRD) [4]. Recognizing the influence of emotional factors on the risk of DM complications, the American Diabetes Association recommends incorporating psychological and social assessments into clinical practice, including screening for DRD, as part of comprehensive care [1, 4].

The DRD refers to the emotional distress resulting from adapting to and living with the disease, and may be associated with the treatment regimen management, the concerns and fears about long‐term outcomes, and the feeling of lack of support from healthcare professionals [5]. This distress tends to be more intense in people at risk of DFD, since factors such as pain, paresthesia, restricted mobility, ulcerations and amputations add to the psychological burdens inherent in managing DM, increasing the emotional impact of the disease [6]. Given this scenario, Primary Health Care (PHC) plays a fundamental role as the entry point for people with DM, providing monitoring, assessment, and prevention of complications, to minimize DRD and promote QoL through access, effectiveness, and comprehensiveness of care [7].

Although DM physical complications have been extensively investigated, knowledge about DRD in people at risk for DFD is still limited. The available evidence focuses predominantly on individuals with already established complications, such as ulcers and amputations, while those at risk for DFD remain understudied, especially within the context of PHC. This gap hinders the understanding of the DRD‐associated factors in the earlier stages of the disease process and the development of preventive strategies targeted at this population.

To address this gap, the study hypothesized that DRD would be associated with sociodemographic and clinical characteristics, as well as factors related to the risk of DFD. Therefore, the objective of the present study was to evaluate DRD and the associated factors in PHC individuals at risk of developing DFD, to provide support for the development of more effective and targeted management strategies, improving QoL and reducing the risk of foot complications in DM people.

## 2 Method

### 2.1 Study Design

This is a cross‐sectional study of factors related to DRD, obtained through data collected at baseline from the cohort entitled “Occurrence of Diabetic Foot and Associated Factors”. The study was conducted with people with DM registered in the urban Family Health Strategy (FHS) in a municipality in the state of Minas Gerais and approved by the Ethics Committee of UFSJ with Opinion Number 6.024.253. The research was designed according to the Strengthening the Reporting of Observational Studies in Epidemiology (STROBE) standards [8]. This study was approved by the ethics committee of the Federal University of São João del‐Rei, under opinion number 4.752.204, and conducted in accordance with the Declaration of Helsinki. Written informed consent was obtained from all participants.

### 2.2 Participants

Participants from 32 urban FHS units were recruited via telephone contact between June 2022 and July 2023, based on their identification in a Brazilian health system database. They were invited to participate and provided with information regarding the main aspects of the study, with any additional questions addressed as needed. Inclusion criteria: Individuals with diabetes mellitus (DM) registered in the Primary Health Care Information System and the Hypertension and Diabetes Registration and Monitoring System of urban Family Health Strategy (FHS) units, aged 18 years or older. Exclusion criteria: individuals receiving care at traditional PHC Centers and those registered with FHS units located in rural areas were excluded because of logistical and communication challenges that could hinder participant recruitment and data collection. Data were obtained through interviews to collect socioeconomic and demographic data, and clinical evaluation with emphasis on examination of the feet and legs for subsequent classification according to risk for DFD was carried out.

Since this is a cross‐sectional phase of a cohort study, the sample size calculation was performed for the occurrence of DFD as the primary outcome. For this purpose, the following was considered: a population of 4826 people with DM and free from DFD, from the 32 FHS Units; significance and confidence levels of 5% and 95% adopted in all statistical inferences, respectively; and statistical power of at least 90% for hypothesis testing about incidence [9]. Based on this power and an incidence of 15% of the outcome (twice that available in the literature) [10], with a margin of error of 2%, the final sample of the study had a final number, *n*_f_ = 1100 individuals. Considering a 30% loss rate, the calculated number of participants was 1430 individuals. In an attempt to reach the largest possible number of participants, given the chance of loss in future follow‐up of the proposed cohort, 1563 people participated in the study.

It should be noted that nine participants were excluded because of missing sociodemographic and clinical data required for the analyses. Glycated hemoglobin values were retrieved from the health information system for participants who had undergone testing within the 3 months preceding data collection. Consequently, HbA1c data were unavailable for a subset of participants, resulting in an analytical sample of 1071 individuals with recorded HbA1c values.

### 2.3 Factors Associated With Diabetes‐Related Distress

The participants' characteristics at the beginning of the study were identified as potential correlates for DRD. These include variables based on general socioeconomic and demographic data. Marital status was categorized as “with a partner” and “without a partner”. Educational status was divided into two categories: lower than (<) finished Elementary Education (indicating participants who did not complete elementary education) and above or equal to (≥) Finished Elementary Education (indicating participants who have completed elementary education or higher). Family income was classified into two ranges: up to 2 and above 2 minimum wages, to assess how different income levels impact the perception of DRD. The age range was divided into two categories: under 60 years old (< 60) and 60 years of age or older (≥ 60), to explore the differences in DRD between younger and older age groups. Moreover, the participants' skin color was classified into two categories: white and non‐white.

### 2.4 Clinical Assessment

The individuals' risk classification for developing DFD was based on the criteria of the International Working Group on the Diabetic Foot [2]. The feet and legs were examined to screen for peripheral arterial disease (PAD) and loss of protective sensation (LOPS). PAD was defined by an ankle‐brachial index (ABI) of less than 0.9 or greater than 1.3, measured using vascular Doppler MEDPEJ. The LOPS was evaluated using a 128 Hz tuning fork and a Semmes‐Weinstein 10 g monofilament, and was considered present when no vibratory or tactile response was observed.

The following risk categories were established: Risk 0 (very low) for individuals without PAD or LOPS; Risk 1 (low) for those with PAD or LOPS; Risk 2 (moderate) for those with both PAD and LOPS, or PAD combined with foot deformities, or LOPS associated with foot deformities; and Risk 3 (high) for individuals with PAD or LOPS, along with a previous history of ulcer, lower limb amputation, or end‐stage renal disease.

Furthermore, the variables related to DM for DRD analysis were specified as follows: time since diagnosis was classified into three groups (less than 5 years, 5–10 years, and more than 10 years); insulin use was recorded based on whether participants use insulin or not; glycemic control was assessed based on HbA1c levels (%), classified as good glycemic control or poor glycemic control; and BMI was divided into non‐overweight and overweight.

Variables related to health behavior were categorized into alcohol consumption (Yes or No) and use of psychotropic drugs (Yes or No). Regarding foot health, the variables were divided as follows: difficulty caring for feet (Yes or No); type of footwear (open sandals or closed sandals); cracked heels (present or not present); interdigital mycosis (present or not present); hypotrophic toenail (present or not present); dry skin (present or not present); calluses (present or not present); and claw toes (present or not present).

### 2.5 Diabetes‐Related Distress Measured Using B‐PAID

The DRD was assessed using the Problem Areas in Diabetes Scale (B‐PAID), a Brazilian version of the Problem Areas in Diabetes (PAID) scale. PAID is a 20‐item screening tool designed to measure the emotional response to diabetes mellitus [11]. Each item is rated on a 5‐point Likert scale, ranging from 0 (not a problem), 1 (minor problem), 2 (moderate problem), 3 (somewhat serious problem), and 4 (a serious problem). The total score, which ranges from 0 to 100, is obtained by adding the answers and multiplying the result by 1.25. A score of 40 or more indicates high DRD.

### 2.6 Statistical Analysis

Data were analyzed using the software *Statistical Package for the Social Sciences* (SPSS), version 26.0. The normality test of the outcome variable (DRD) was performed using the Shapiro–Wilk test and QQNorm plot, both of which indicating a non‐normal distribution. Descriptive analysis was applied to establish the participants' characteristics and to define their proportion with a PAID score ≥ 40. Considering the asymmetrical distribution of the outcome (DRD) and given that it consists of positive values, Gamma regression models were used. This model follows the same logic as other generalized linear models. The results for the variables are adjusted for the others, meaning there is no confounding among the modeled variables. Regression was performed with the simultaneous input of all predictors to examine the effect of each one when adjusting for the variance shared among them. All variables were entered simultaneously using a criterion of 0.05 for variable entry and 0.10 for removal. Thus, from the model with all variables, those significant at a 5% level were selected, composing the final model with sex, age group, insulin use, glycated hemoglobin, difficulty in foot care, LOPS, and BMI. The qualitative variables were categorized according to previously established clinical and epidemiological criteria defined by the Brazilian Institute of Geography and Statistics and the Brazilian Association for the Study of Obesity and Metabolic Syndrome [12, 13]. Although this approach may reduce statistical power, it enhances the clinical and sociodemographic interpretability of the findings. To address potential limitations associated with this categorization, the statistical model was designed to optimize model fit and minimize potential interpretative bias.

## 3 Results

This study had 1563 participants who met the eligibility criteria and were included in the analysis. The participants' characteristics are detailed in Table 1. Thus, through descriptive analysis, the median of DRD was 16 (27), which suggests a varied distribution in the participants' perceptions. Regarding the risk of developing DFD, it was observed that a significant portion of the participants had no risk of complications (60.2%). However, approximately 39.8% of individuals were identified as being at some level of risk.

**TABLE 1:** Factors related to diabetes‐related distress.

Variables	*N* (%)	Median (IQR)	*p value*
Quality of life^a^ Median (IQR)		16 (27)	
Sex			
Male	989 (63.3%)	9 (21)	< 0.001
Female	574 (36.7%)	20 (28)	
Age range			
< 60	589 (37.7%)	22 (29)	< 0.001
≥ 60	974 (62.3%)	12 (25)	
Color			
White	606	15 (25.25)	
Not white	952	16 (29)	0.048
Marital status			
With a partner	948 (60.7%)	16 (28)	0.019
Without a partner	615 (39.3%)	17 (25)	
Level of education			
< finished elementary education	925 (59.2%)	16 (27)	0.247
≥ finished elementary education	638 (40.8%)	16 (27)	
Income			
Up to 2 minimum wages	718 (45.9%)	17 (29)	0.064
Above 2 minimum wages	845 (54.1%)	15 (26)	
Occupation			
Active	443 (28.3%)	18 (25)	0.796
Inactive	1120 (71.7%)	15.5 (28)	
Alcoholic beverage			
Yes	369 (23.6%)	14.5 (26)	0.006
No	1194 (76.4%)	16 (27)	
Diagnostic time of DM			
Less than 5 years ago	378 (24.2%)	16 (27)	0.053^b^
5–10 years of illness	426 (27.3%)	16 (26.75)	
More than 10 years	759 (48.6%)	16 (28)	
Use of insulin			
Yes	466 (29.8%)	24 (34)	< 0.001
No	1097 (70.2%)	14 (24)	
Psychotropic drugs			
Yes	347 (22.2%)	21 (28.5)	< 0.001
No	1202 (76.9%)	16 (27)	
Difficulty with foot care			
Yes	689 (44.1%)	17 (30)	0.001
No	874 (55.9%)	16 (25)	
Type of footwear			
Open sandal	1163 (74.4%)	16 (28)	0.023
Closed sandal	400 (25.6%)	14.5 (23)	
Cracks			
Presence	506 (32.4%)	17 (28)	0.016
Absent	1057 (67.6%)	16 (27)	
Interdigital mycosis			
Presence	304 (19.4%)	16 (27)	0.501
Absent	1259 (80.6%)	16 (27)	
Claw‐like fingers			
Presence	87 (5.6%)	9 (28)	0.374
Absent	1476 (94.4%)	16 (26)	
Charcot arthropathy			
Presence	53 (3.4%)	20 (33.75)	0.336
Absent	1510 (96.6%)	16 (27)	
Suspected PAD			
Suspect	1006 (64.4%)	16 (27)	0.073
No suspicion	557 (35.6%)	16 (30)	
DF risk			
Very low risk	941 (60.2%)	16 (25)	0.021^b^
Low risk	492 (31.5%)	17 (28)	
Moderate risk	100 (6.4%)	17.5 (38.50)	
High risk	30 (1.9%)	16 (46.50)	
LPS			
Present	180 (11.5%)	28 (34)	< 0.001
Absent	1383 (88.5%)	16 (27)	
BMI			
No overweight	1022 (65.4%)	14 (24)	< 0.001
Overweight	541 (34.6%)	18 (29)	
HbA1c %			
Good control	542 (34.7%)	12.5 (24)	< 0.001
Poor control	529 (33.8%)	19 (29)	

*Note:* Frequency (%); Glycated Hemoglobin (HbA1c%); Age range (< under 60 years old; 60 years old or older); Education (< lower than finished elementary education; ≥ greater than or equal to finished elementary education); Income (≤ less than or equal to 2 minimum wages; > greater than 2 minimum wages).

Abbreviations: BMI: Body Mass Index; LPS: Loss of Protective Sensation; PAD: Peripheral Arterial Disease.

^a^ Median (Interquartile Range).

^b^ Kruskal–Wallis test.

The factors observed after the bivariate analyses were determined and included in the multivariate analyses. Individuals at risk of developing DFD showed higher DRD (median = 17; IQR = 30) compared to those without risk. Furthermore, insulin use was strongly associated with an increase in DRD (median = 24; IQR = 34) in relation to non‐users, with *p* < 0.001. Glycemic control also stood out, with individuals with poor control showing higher levels of DRD (median = 19; IQR = 29) compared to those with good control, with *p* < 0.001.

The results presented in Table 2, obtained through the multiple Gamma regression model, indicate that women, individuals younger than 60 years, insulin users, those reporting difficulties with foot care, individuals with loss of protective sensation (LOPS), and those who were overweight had higher DRD scores than their respective reference groups. In contrast, participants with good glycaemic control had lower DRD scores than those with poor glycaemic control (reference), indicating that poor glycaemic control was associated with greater diabetes‐related distress.

**TABLE 2:** Final multiple regression model of factors associated with diabetes‐related distress score.

Variables	Exponential of beta (CI)	*p* value
Sex (Male)	0.795 (0.712–0.88)	< 0.001
Sex (Female)	—	—
Age range (< 60)	1.309 (1.175–1.475)	< 0.001
Age range (≥ 60)	—	—
Use of insulin (yes)	1.211 (1.071–1.370)	0.002
Use of insulin (no)	—	—
Difficulty with foot care (yes)	1.129 (1.014–1.257)	0.026
Difficulty with foot care (no)	—	—
LPS (present)	1.311 (1.110–1.549)	0.001
LPS (absent)	—	—
BMI (overweight)	1.161 (1038–1.298)	0.009
BMI (no overweight)	—	—
HbA1c% (good control)	0.850 (0.761–0.951)	0.004
HbA1c% (poor control)	—	—

Abbreviations: BMI: Body Mass Index; HbA1c%: Glycated hemoglobin; LPS: Loss of Protective Sensation.

## 4 Discussion

The results of this study reveal important associations between clinical and behavioral factors and the DRD of individuals in PHC at risk of developing DFD. In general, the higher scores, which indicate an increase in DRD, were significantly associated with being a woman, being an adult under 60 years old, insulin use, difficulties caring for the feet, LOPS, overweight, and inadequate HbA1c control. In addition, evidence suggests that interventions targeting individuals at risk of DFD should address not only clinical aspects and self‐care behaviors, but also DRD, since more threatening perceptions of the disease can negatively influence the adoption and maintenance of these practices [5]. Accordingly, DRD can represent an important mechanism in the relationship between the way an individual experiences the risk of complications and their engagement in self‐care, reinforcing the need for comprehensive approaches in the PHC.

This study revealed that being a woman was associated with higher levels of DRD. Women presented significantly higher scores when compared to men, indicating a greater emotional impact resulting from the disease. This finding is consistent with previous studies that indicate that women at risk of DFD tend to experience higher levels of disease‐related distress, possibly due to a greater burden of social and family roles, a higher prevalence of anxiety and depressive symptoms, and differences in the perception and coping with treatment demands [14]. Similarly, Chew et al. observed an association between DRD and depressive symptoms, suggesting that the higher prevalence of depression among women, approximately twice that observed among men, may represent an underlying, often undiagnosed factor, contributing to the higher levels of psychological distress found in this group [15]. These results reinforce the need for attention to the emotional aspects of women with DM and at risk of DFD, favoring the early identification of DRD in PHC.

Higher DRD scores were observed in individuals who use insulin. The observed association can be explained by the fact that more complex therapeutic management, such as insulin therapy, tends to impose greater demands for self‐care and a greater emotional burden [16]. The need for insulin therapy can be interpreted as a sign of disease progression, triggering feelings of anxiety, sadness and insecurity related to the treatment demands [4, 17]. Furthermore, studies indicate that individuals using insulin exhibit greater emotional impairment, possibly due to the treatment requirements, such as the need for multiple daily injections and greater rigor in self‐management [17].

People who reported difficulties in foot care obtained higher scores on DRD compared to those who do not face these challenges, showing that difficulty in self‐care is associated with greater emotional distress and reinforcing the importance of this practice in managing the risk of DFD [18]. Physical limitations, such as back pain, knee pain, and increased waist circumference, can hinder the ability of bending down and seeing the feet, impairing the capacity of carrying out the necessary care. Early recognition of foot problems may contribute to reducing complications associated with DM, as well as decreasing morbidity and mortality [19]. However, although risk perception may encourage the adoption of preventive behaviors, high levels of DRD can compromise engagement in self‐care, hampering the maintenance of recommended practices for the prevention of DFD [5]. Thus, adopting a regular self‐care routine constitutes the most effective primary strategy to prevent foot complications and promote health, contributing to the reduction of DRD [20]. Within this framework, the relevance of the nurse's role in PHC is understood, with a view to supporting measures for self‐care, as well as for correct assessment, adoption of conduct for effective treatment and health education, aiming at adequate care in prevention [21].

LOPS was another factor significantly associated with a worse score related to DRD, with a substantial increase compared to individuals without LOPS. This result underscores evidence that LOPS, besides representing an important risk factor for the development of foot ulcers, may be associated with higher DRD, possibly due to concerns about disease progression, loss of functionality, and the occurrence of future complications [22]. In this regard, screening for LOPS can play a critical role for this purpose [23]. Experts from international and national organizations suggest that people with DM undergo systematic screenings and examinations of the feet at least once a year, increasing the frequency in cases of arterial and/or sensory disorders [24, 25]. Timely referral of people with severe clinical signs and symptoms ensures appropriate treatment at the right time, which contributes significantly to improved clinical outcomes [26]. Nevertheless, despite its importance in monitoring these individuals, PHC does not always have the necessary resources to identify and address factors associated with DRD and the risk of DFD early on, which can compromise the comprehensiveness of care [25].

Being overweight was associated with a higher score compared to individuals with normal weight, suggesting that excess weight may worsen DRD among individuals and increase the risk of developing DFD. The literature documents that a high BMI in overweight people increases the chances of developing DFD almost fivefold [27]. Furthermore, the link between BMI and DM is related to insulin resistance, oxidative stress, mitochondrial homeostasis dysregulation and other factors, and this effect often extends beyond the usual clinical observation period [28, 29]. A meta‐analysis indicated that a BMI above 24.5 represents an independent risk factor for the occurrence of foot ulcers [30]. Therefore, altered BMI in people with DM can lead to significant clinical and psychosocial repercussions, including reduced life expectancy, a higher incidence of comorbidities, DRD enhancement, and increased risk of developing DFD [27, 28]. These findings reinforce the importance of effective PHC interventions focused on weight management and promotion of healthy lifestyles, combined with psychosocial support. On this basis, longitudinal care and the bond established between professionals and users favor the early identification and management of modifiable risk factors, contributing to the prevention of complications and to the provision of care centered on the individual at risk of DFD [31].

The findings regarding inadequate glycemic control deserve attention, since DRD can compromise the adoption of behaviors essential for managing DM. Individuals with higher levels of distress tend to have lower adherence to physical activity, dietary plans, and medication treatment, favoring inadequate glycemic control [32]. Conversely, the persistence of high glycemic levels can also intensify concerns and demands related to treatment, establishing a cycle that enhances DRD [33]. Corroborating these findings, the American Diabetes Association recommends routine screening for depressive disorders and other psychosocial aspects in people at risk of foot complications, especially those who have difficulty meeting treatment goals, recognizing the impact of these factors on self‐care and clinical outcomes [33, 34]. However, because it often remains hidden in clinical practice, DRD still represents a challenge for healthcare professionals, as it can be underdiagnosed and exacerbate difficulties in managing DFD, with negative repercussions on glycemic control and DRD in these individuals [35].

In this context, PHC has the potential to incorporate care strategies that transcend traditional biomedical management, including approaches focused on emotional support and strengthening of the coping capacities of individuals with DM and at risk of DFD [36]. Unlike interventions focused exclusively on health education or drug treatment, these strategies are based on a therapeutic relationship of trust, qualified listening, and continuous support, promoting changes in the emotional, cognitive, and behavioral aspects involved in disease management. By promoting autonomy, empowerment, and greater confidence in self‐care, such interventions can strengthen self‐management of the chronic condition, improve adherence to therapeutic recommendations, and contribute to better clinical and psychosocial outcomes [37]. Thus, by analyzing attributes such as access, longitudinality, comprehensiveness, and bonding, PHC is configured as a strategic space for the recognition and management of DRD, contributing to the prevention of complications related to DFD.

The limitations of this study include its cross‐sectional design, since the findings should be interpreted as associations, without allowing causal inferences. Moreover, comorbidities and life events potentially related to DRD were not evaluated, which may have influenced the observed results. Considering the scarcity of studies on DRD in people with or at risk of DFD, further research is required to broaden the understanding of this phenomenon at different stages of foot complications in people with DM and to support the development of more targeted and effective interventions within the scope of PHC.

This study highlights important points, including a large and representative sample of individuals with DM followed up in PHC, as well as the use of standardized measures specific to diabetes, which facilitate the replication of results. Additionally, the findings provide previously unexplored evidence regarding DRD in people at risk of DFD, contributing to the advancement of knowledge in this area. The convergence of the results with evidence already available in the literature gives greater impact to the findings and reinforces the need to incorporate the evaluation of DRD during comprehensive care to this population.

## 5 Conclusion

Associations with sociodemographic and clinical variables can guide PHC in planning care for people with DFD. In this regard, it should be noted that higher distress is observed in women, in patients on insulin therapy, those with foot care difficulties, those who are overweight, and those with poor glycemic control.

These groups require longitudinal and individualized follow‐up, with the routine incorporation of DRD assessment as part of comprehensive care. Strategies that integrate education for self‐care, support for insulin therapy management, prevention of foot complications, promotion of weight control, and optimization of glycemic control, combined with psychosocial support, can contribute to minimizing DRD, strengthening engagement in treatment, and promoting better health outcomes.

## Author Contributions

**Thallita Cláudia Moraes Barbosa:** conceptualization, investigation, methodology, visualization, writing – original draft, preparation, writing – review and editing. **Letícia Eugênio Mota:** investigation, visualization, writing – review and editing. **Maria Gabriela Campos Nunes:** investigation, visualization, writing – review and editing. **Laura Andrade Pinto:** investigation, visualization, writing – review and editing. **Letícia Alves Rocha:** investigation, visualization, writing – original draft, writing – review and editing. **Patricia Peres de Oliveira:** validation, visualization, writing – review and editing. **Vinicius Silva Belo:** formal analysis, methodology, supervision, writing – review and editing. **Daniel Nogueira Cortez:** conceptualization, formal analysis, funding acquisition, investigation, methodology, project administration, supervision, visualization, writing – original draft, preparation, writing – review and editing.

## Funding

This work was funded by the Minas Gerais State Research Support Foundation (FAPEMIG) (APQ‐04289‐22), National Council for Scientific and Technological Development (CNPq) (Grant 420227/2023‐7).

## Ethics Statement

The study was approved by the Ethical Committee from Federal University of São João del‐Rei (4.752.204).

## Consent

Informed consent was obtained from all individual participants included in the study.

## Conflicts of Interest

The authors declare no conflicts of interest.

## Data Availability

The data that support the findings of this study are available from the corresponding author upon reasonable request.
